# A nomogram for early risk stratification of 30-day mortality in confirmed *Acinetobacter baumannii* bloodstream infection

**DOI:** 10.3389/fcimb.2026.1979847

**Published:** 2026-10-30

**Authors:** Lingyao Ma, Zhenzhen Cai, Jingping Liu, Ning Dong, Jun Zhou, Jian Xu

**Affiliations:** 1 Department of Laboratory Medicine, The First Affiliated Hospital with Nanjing Medical University, Nanjing, China; 2 Branch of National Clinical Research Center for Laboratory Medicine, Nanjing, China; 3 Department of Quality Management, The First Affiliated Hospital with Nanjing Medical University, Nanjing, China

**Keywords:** 30-day mortality, *Acinetobacter baumannii*, bloodstream infection, LASSO regression, nomogram

## Abstract

**Purpose:**

*Acinetobacter baumannii* bloodstream infection (BSI) carries high mortality. This study aimed to develop and validate a nomogram incorporating age and routine laboratory parameters to predict 30-day mortality in patients with confirmed *A. baumannii* BSI, before antimicrobial susceptibility results are available.

**Methods:**

This single-center retrospective study split into training (n=202) and validation (n=87) cohorts. Demographic data (sex, age) and laboratory parameters at diagnosis were collected for model development, with the aim of providing early risk stratification after confirmation of *A. baumannii* BSI but before susceptibility results are available. Clinical characteristics were recorded for baseline description only. LASSO regression, the Boruta algorithm and multivariate logistic regression were employed to identify independent predictors from sex, age, and all laboratory indicators.

**Results:**

The overall 30-day mortality rate was 33.17%. Five independent risk factors were identified: older age, elevated blood urea nitrogen (Urea), elevated sodium (Na), prolonged activated partial thromboplastin time (APTT), and lower mean corpuscular hemoglobin concentration (MCHC). The nomogram incorporating these factors demonstrated good discrimination, with an area under the ROC curve of 0.813 in the training cohort and 0.808 in the validation cohort. Calibration and decision curve analyses confirmed its accuracy and clinical utility.

**Conclusion:**

The developed nomogram may serve as a potential tool for predicting 30-day mortality in patients with confirmed *A. baumannii* BSI, before antimicrobial susceptibility results are available. It is intended to support early risk assessment rather than to guide empirical antibiotic selection, which requires further prospective validation.

## Introduction

*Acinetobacter baumannii* (*A. baumannii*) is a Gram-negative opportunistic pathogen within the genus *Acinetobacter*, which comprises over 50 species (Al Atrouni et al., 2016). It has emerged as a leading cause of healthcare-associated infections (HAIs) worldwide. In the United States and Europe, *A. baumannii* accounts for approximately 2% of all HAIs (Magill et al., 2014; Lob et al., 2016), whereas in Asia and the Middle East, its prevalence is roughly twice as high (Giammanco et al., 2017). Bloodstream infection (BSI) is defined by positive blood cultures in a patient presenting with systemic signs of infection and may be either secondary to a documented source or primary (i.e., without an identified source) (Timsit et al., 2020). Due to factors such as increased antibiotic exposure, frequent invasive procedures, and the rise of carbapenem resistance, *A. baumannii* has become one of the most prevalent pathogens causing BSI in critically ill patients (Qian et al., 2022). It is responsible for an estimated 9%–35% of all BSIs, a proportion that continues to increase (Wang et al., 2018). The overall mortality rate for patients with *A. baumannii* BSI ranges from 29% to 63% (Nutman et al., 2014; Gu et al., 2016). Consequently, *A. baumannii* BSI represents a major clinical challenge, and timely, accurate etiological diagnosis combined with early, adequate antimicrobial therapy are crucial for improving patient outcomes.

Traditional blood culture (BC) methods suffer from notable limitations, including low sensitivity, prolonged turnaround times, and susceptibility to prior antibiotic use. Currently, comorbidities, APACHE II scores, immunosuppressive status, and the timeliness of antimicrobial therapy selection are recognized as prognostic factors influencing *A. baumannii* BSI (Niu et al., 2018; Huang et al., 2020; Wei et al., 2024). Additionally, clinical risk factors such as procalcitonin (PCT) levels can guide diagnostic and therapeutic decision-making, with digital PCR (dPCR) offering potential enhancements in detection accuracy (Lin et al., 2025). Nevertheless, existing studies exhibit limitations, leaving a need for a standardized and clinically feasible prognostic prediction model. A nomogram is a mathematical tool that predicts disease progression or mortality by integrating weighted prognostic parameters derived from patient outcome analyses (Iasonos et al., 2008). Its utility in disease diagnosis and prognosis has expanded significantly in recent years. To address the clinical challenges posed by *A. baumannii* BSI, this study analyzed demographic and routine laboratory data to develop and validate a nomogram predicting 30-day mortality risk. This tool facilitates early identification of high-risk patients, enabling timely interventions to improve outcomes.

## Methods

### Study design and participants

This was a retrospective cohort study. We collected clinical data from patients aged 18 years or older who were diagnosed with *A. baumannii* BSI at the First Affiliated Hospital With Nanjing Medical University between January 2017 and July 2025.

The inclusion criteria were as follows:

Patients diagnosed with *A. baumannii* BSI, meeting both of the following conditions:

1. Fulfilling the clinical diagnostic criteria for BSI: fever (>38 °C) or hypothermia (<36 °C), which may be accompanied by chills, plus at least one of the following: ① Invasive portal venous lesions or migratory lesions; ② Systemic toxic signs without an obvious focus of infection; ③ Skin rashes, petechiae, or hepatosplenomegaly, or neutrophilia with nuclear left shift in peripheral blood that cannot be otherwise explained; ④ Systolic blood pressure <12 kPa (90 mmHg) or a reduction of >5.3 kPa (40 mmHg) from the baseline systolic blood pressure.
2. At least one peripheral venous blood culture result is positive for *A. baumannii* (skin colonization contamination should be excluded, meeting any of the following conditions: ① Two blood cultures taken at different time points isolate the same strain of *A. baumannii*; ② When blood culture is positive, *A. baumannii* of the same source is cultured from other infection sites (such as respiratory tract, urinary tract, wound); ③ After the blood culture is positive, antimicrobial therapy is effective and clinical infection symptoms are alleviated).
3. Age≥18 years at the time of diagnosis.
4. Availability of complete data on overall survival.
5. For patients with multiple *A. baumannii*-positive blood cultures, data from the first positive culture were used.

The exclusion criteria were as follows:

1. Hospital stay < 48 hours.
2. BSI caused by multiple microorganisms (polymicrobial BSI), not possible to clearly identify *A. baumannii* as the primary pathogen.
3. Blood culture results are contaminated (e.g., single positive culture, no clinical signs of infection, no homologous strains at other sites, and negative on repeated cultures).
4. Incomplete documentation of clinical data.

Ultimately, our study comprised a sample of 289 patients. Patients were randomly assigned to a training cohort (n=202) and a validation cohort (n=87) at a 7:3 ratio, stratified by 30-day mortality status to ensure balanced outcome distribution between cohorts. The study protocol was approved by the Ethics Committee of the First Affiliated Hospital of Nanjing Medical University (Nanjing, China). This research adheres to the principles outlined in the Helsinki Declaration. The Institutional Review Board of the First Affiliated Hospital With Nanjing Medical University waived the requirement for informed consent, given the anonymous nature of the retrospective data and the non-interventional study design, Clinical trial number: not applicable.

### Data acquisition and feature selection

Data for this study were extracted from the electronic medical record system. A total of 88 variables were initially collected for baseline characterization. However, feature selection for the prediction model was restricted to sex, age, and all laboratory parameters.

1. Baseline Characteristics: This category comprised demographic information (sex, age), past medical history, history of blood transfusion, surgical history, length of hospital stay, intensive care unit (ICU) admission status, complications (e.g., septic shock, organ failure), use of hormones or immunosuppressants, and exposure to invasive procedures (e.g., invasive mechanical ventilation, venous/arterial catheterization, and placement of drainage tubes, urinary catheters, or gastrointestinal tubes), as well as renal replacement therapy and multidrug resistant bacterial infection.
2. Laboratory Indicators: Laboratory indicators were collected on the day of blood culture sampling or within the subsequent 24-hour period, covering the following three subcategories: 1) Routine Blood Test Parameters: Including C-reactive protein (CRP), white blood cell (WBC) count, lymphocyte (LY) count, monocyte (MO) count, neutrophil (NE) count, lymphocyte percentage (LY%), monocyte percentage (MO%), neutrophil percentage (NE%), red blood cell (RBC) count, hemoglobin (HGB) level, hematocrit (HCT), mean corpuscular volume (MCV), mean corpuscular hemoglobin (MCH), mean corpuscular hemoglobin concentration (MCHC), red blood cell distribution width (RDW), platelet (PLT) count, plateletcrit (PCT), mean platelet volume (MPV), and platelet distribution width (PDW). 2) Coagulation Markers: Prothrombin time (PT), international normalized ratio (PT-INR), activated partial thromboplastin time (APTT), fibrinogen (FIB), thrombin time (TT), and D-dimer. 3) Biochemical Parameters: These were further divided into: Liver and Kidney Function (Alanine aminotransferase (ALT), aspartate aminotransferase (AST), alkaline phosphatase (ALP), γ-glutamyl transpeptidase (GGT), creatine kinase (CK), lactate dehydrogenase (LDH), blood urea nitrogen (BUN), serum creatinine (CREA), uric acid (UA), total bilirubin (TBIL), direct bilirubin (DBIL), and indirect bilirubin (IBIL)). Serum Electrolytes (Potassium (K), sodium (Na), chloride (Cl), calcium (Ca), phosphorus (P), and magnesium (Mg)). Lipid Profiles (Total cholesterol (TC), triglycerides (TG), high-density lipoprotein cholesterol (HDL-C), low-density lipoprotein cholesterol (LDL-C), and lipoprotein(a) (Lp(a))). Protein-Related Parameters (Total protein (TP), albumin (ALB), and globulin (GLB)). Of the 88 variables initially collected, 48 clinical characteristics were excluded *a priori* from model development—retained only for baseline description—because the aim of this study was to build a prediction model based primarily on routine laboratory parameters. Among the remaining 40 demographic and laboratory candidates, variables with >10% missingness were excluded, and random forest imputation was applied to those with ≤10% missingness. All variables were kept on their original measurement scales; no standardization was applied. Collinearity among the candidate variables was assessed using variance inflation factors (VIF), and variables with VIF > 5 were considered to have significant collinearity and were excluded or merged.

### Feature selection from sex, age, and laboratory parameters

Given the limited sample size (n = 202 in the training cohort) and the relatively large number of candidate variables, a single feature-selection method may yield unstable or overfitted results. We therefore applied two feature-selection methods (LASSO regression and the Boruta algorithm), and retained only the intersection of variables selected by both. This strategy was intended to identify the most stable and parsimonious set of predictors, reduce the risk of false positives from either method alone, and mitigate overfitting. Variables identified by both algorithms were retained as candidate features. Subsequently, multivariate logistic regression was performed on these candidates to select independent predictors of 30-day mortality, estimate independent odds ratios and 95% confidence intervals, assess collinearity via variance inflation factors (VIF), and provide regression coefficients for nomogram construction.

### Follow-up

The primary endpoint was predefined as all-cause mortality within 30 days following *A. baumannii* BSI diagnosis. Mortality data were collected from medical records and by telephone call.

### Statistical analysis

Statistical analyses were performed by IBM SPSS version 21.0 (SPSS Inc., Chicago, IL, USA) and R (4.3.1). Quantitative variables are expressed as medians with interquartile ranges (IQRs)/mean ± standard deviation (SD) and were assessed by the Mann-Whitney U test, the Kruskal–Wallis H test or the Student’s t-test. The Chi-square test was employed to analyze categorical variables. LASSO, Boruta algorithm and multivariate regression analysis were applied for identifying potential and independent risk factors. At last, a nomogram was constructed based on the multivariate regression analysis. In addition, the discrimination performance of the model was evaluated by using the area under the receiver operating characteristic (ROC) curve (AUC), as well as sensitivity and specificity. P<0.05 was considered statistically significant.

## Results

### Baseline characteristics and clinical profiles

A total of 289 patients with *A. baumannii* BSI were enrolled in this study. They were randomly assigned to a training cohort (n = 202) and a validation cohort (n = 87) at a ratio of 7:3. No significant differences between the validation and training cohorts (Except RBC and HCT), indicating good consistency between the two cohorts (**Table 1**; **Supplementary Table 1**). The flowchart of the study design is presented in **Figure 1**.

**Table 1:** Baseline characteristics and laboratory parameter.

Characteristic	Training cohort				Validation Cohort N = 87	P_2-_value
	Overall N = 202	Survivor N = 135	Non-survivor N = 67	P_1_-value		
Gender(male), n (%)	132 (65.3%)	84 (62.2%)	48 (71.6%)	0.185	62 (71.3%)	0.326
Age, y, M (Q1, Q3)	63 (50, 70)	57 (49, 66)	69 (57, 77)	<0.001	66 (50, 73)	0.102
MCHC (g/L), M (Q1, Q3)	327 (317, 336)	329 (321, 337)	321 (309, 331)	<0.001	328 (316, 338)	0.582
PLT(×10^9^/L), M (Q1, Q3)	129 (63, 200)	140 (77, 232)	88 (47, 151)	<0.001	123 (68, 173)	0.882
Pro-BNP (pg/mL), M (Q1, Q3)	2,617 (706, 7,497)	2,237 (554, 5,541)	4,229 (872, 10,787)	0.003	2,401 (723, 6,619)	0.850
PCT (ng/mL), M (Q1, Q3)	2 (0, 8)	2 (0, 8)	4 (1, 11)	0.039	2 (0, 6)	0.250
PT (s), M (Q1, Q3)	14.3 (12.7, 17.1)	13.7 (12.6, 15.9)	16.4 (13.8, 19.4)	<0.001	14.2 (13.3, 15.9)	0.669
PT-INR, M (Q1, Q3)	1.29 (1.13, 1.59)	1.22 (1.11, 1.43)	1.51 (1.25, 1.85)	<0.001	1.27 (1.18, 1.47)	0.741
APTT (s), M (Q1, Q3)	34 (29, 44)	31 (29, 38)	41 (32, 54)	<0.001	34 (30, 41)	0.967
TT (s), M (Q1, Q3)	16.8 (15.6, 18.3)	16.6 (15.6, 17.8)	17.5 (15.8, 19.2)	0.037	17.3 (15.7, 18.7)	0.487
GGT (U/L), M (Q1, Q3)	59 (29, 163)	67 (32, 192)	44 (23, 103)	0.017	68 (28, 126)	0.779
LDH (U/L), M (Q1, Q3)	320 (236, 417)	302 (220, 383)	331 (267, 534)	0.003	300 (220, 421)	0.704
HBDH (U/L), M (Q1, Q3)	197 (147, 268)	190 (127, 252)	216 (169, 351)	0.001	186 (124, 280)	0.306
TC (mmol/L), M (Q1, Q3)	2.85 (1.78, 3.77)	2.97 (2.09, 3.94)	2.03 (1.46, 3.16)	<0.001	2.53 (1.78, 3.33)	0.394
TP (g/L), Mean ± SD	58 ± 9	59 ± 8	55 ± 9	0.001	58 ± 7	0.776
Urea (mmol/L), M (Q1, Q3)	9 (6, 16)	8 (6, 13)	13 (8, 23)	<0.001	11 (7, 19)	0.231
Na (mmol/L), M (Q1, Q3)	140 (137, 144)	140 (136, 142)	143 (140, 146)	<0.001	140 (137, 144)	0.722

APTT, Activated Partial Thromboplastin Time; GGT, Gamma-Glutamyl Transferase; HBDH, Hydroxybutyrate Dehydrogenase; LDH, Lactate Dehydrogenase; MCHC, Mean Corpuscular Hemoglobin Concentration; PCT, Procalcitonin; PLT, Platelets; Pro-BNP, Pro-B-type Natriuretic Peptide; PT, Prothrombin Time; PT-INR, Prothrombin Time International Normalized Ratio; TC, Total Cholesterol; TP, Total Protein; TT, Thrombin Time.

**Figure 1:**
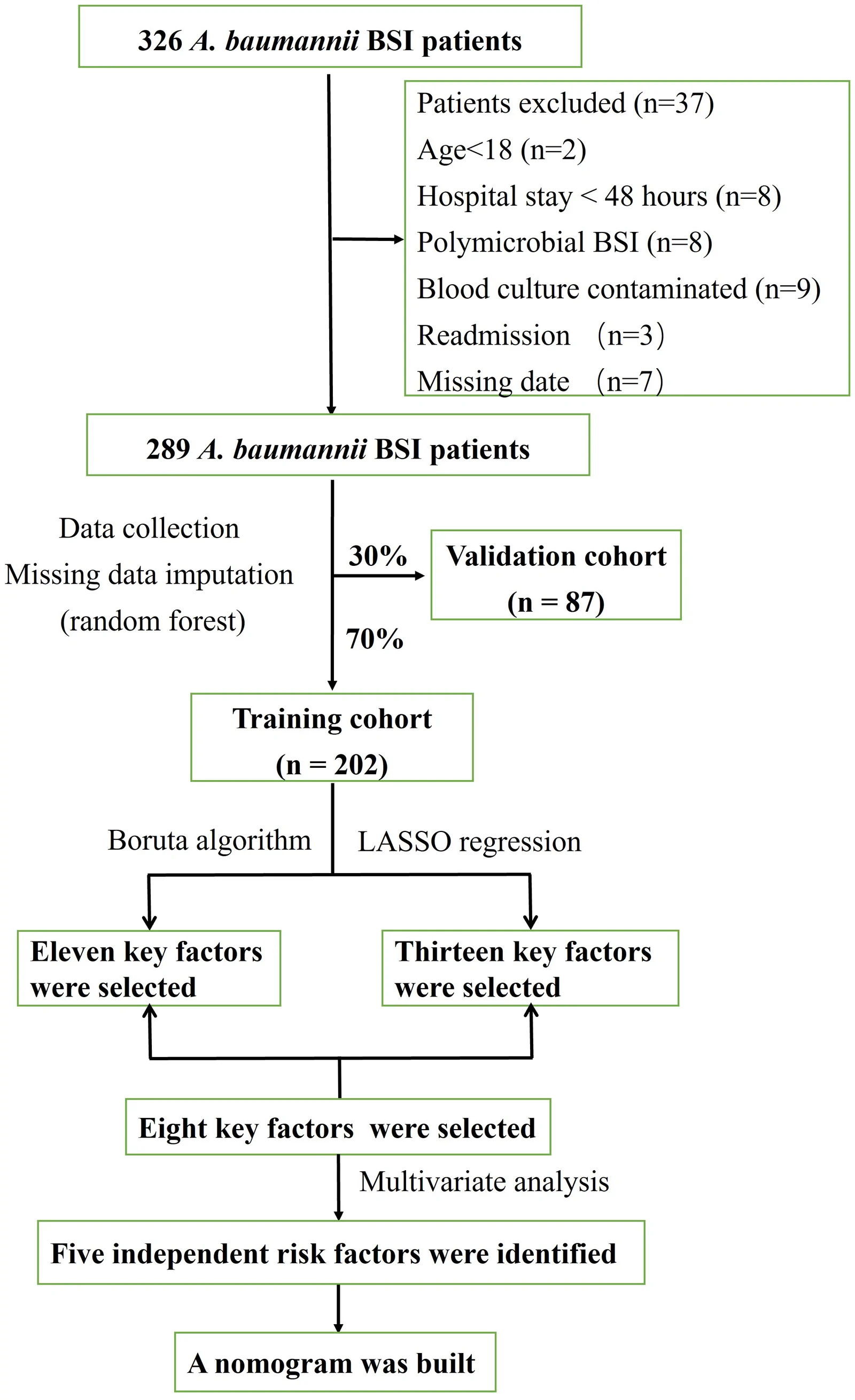
Flow chart of the survey design.

In the training cohort (n = 202), patients were categorized into survivors (n=135) and non-survivors (n=67) based on their 30-day survival status, resulting in an overall mortality rate of 33.17%. Comparison of clinical characteristics revealed statistically significant differences between the two groups in age, history of diabetes, cardiovascular disease, respiratory disease, prior surgery, invasive mechanical ventilation, deep vein catheterization, gastrointestinal catheterization, renal replacement therapy, septic shock, organ failure, multidrug resistant bacterial infection and use of hormones or immunosuppressants (all p < 0.05).

Laboratory parameters also differed significantly between the groups. EO count, EO percentage, RBC, HGB, MCHC, PLT, PCT, FIB, GGT, TC, HDL, LDL, TP, ALB, GLB, and P levels were significantly higher in the survival group than in the non-survival group (p < 0.05). In contrast, the values of MCV, RDW, MPV, pro-BNP, PCT, PT, PT-INR, APTT, TT, LDH, HBDH, GLU, Urea, Cr, Na in non-survival group were significantly higher than those in survival group (all p<0.05). No significant differences were observed for the remaining covariates (all p > 0.05) (**Table 1**). The baseline characteristics of all variables are detailed in **Supplementary Table S1**.

### Prediction of risk factors based on LASSO regression and Boruta algorithm

Given the limited sample size, LASSO regression and Boruta were employed to select predictive variables. Under the optimal lambda value, 13 predictive variables with non-zero coefficients were selected: age, Urea, PT-INR, Na, APTT, TT, HBDH, pro-BNP, GGT, PLT, MCHC, TC, TP through LASSO regression. 11 predictive variables were selected: age, Urea, PT, PT-INR, Na, APTT, LDH, PCT, HBDH, PLT, MCHC through Boruta algorithm. The intersection of variables identified by both methods was taken, resulting in a combined set of 8 predictive variables (**Figure 2**). Before entering multivariate logistic regression, collinearity among these eight variables was assessed using variance inflation factors (VIF). The VIF values were 1.054 for age, 1.069 for Urea, 1.056 for Na, 1.047 for MCHC, 1.104 for PLT, 1.422 for PT-INR, 1.567 for APTT, and 1.126 for HBDH. All values were below 2, indicating no significant collinearity. Subsequent multivariate logistic regression identified five independent risk factors for 30-day mortality in *A. baumannii* BSI: age (OR = 1.057, 95% CI: 1.030–1.085, p < 0.001), Urea (OR = 1.050, 95% CI: 1.011–1.092, p = 0.013), Na (OR = 1.057, 95% CI: 1.003–1.113, p = 0.037), MCHC (OR = 0.990, 95% CI: 0.981–0.999, p = 0.038), and APTT (OR = 1.048, 95% CI: 1.018–1.079, p = 0.002) (**Table 2**).

**Figure 2:**
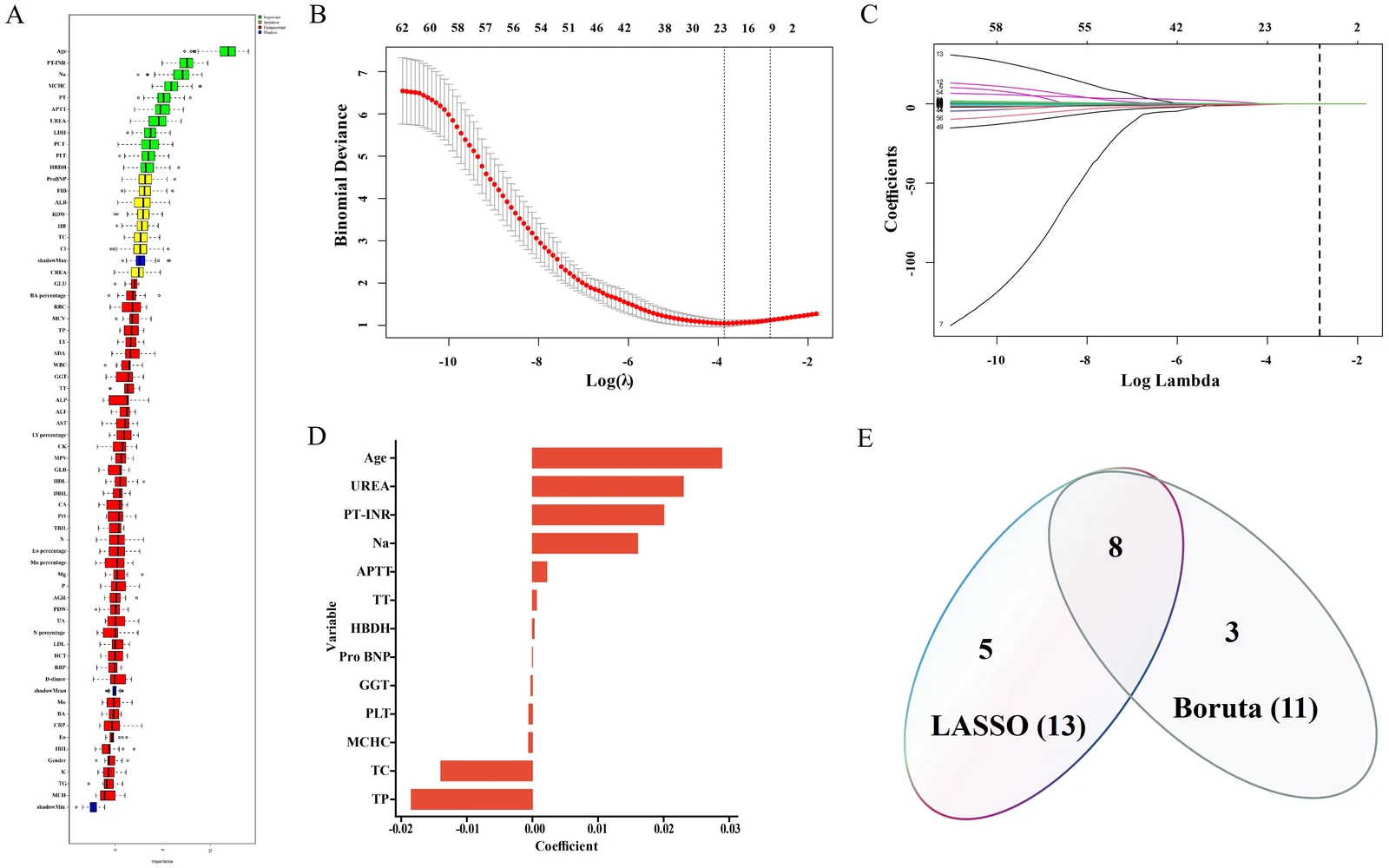
Screening of variables based on Boruta algorithm and LASSO regression. **(A)** Boruta algorithm. **(B)** LASSO regression cross-validation curve. **(C)** The coefficient trajectory plot of LASSO. **(D)** The feature coefficient graph after LASSO selection parameters. **(E)** Intersection graph of LASSO regression and Boruta algorithm feature selection methods.

**Table 2:** Multivariate logistic regression analysis of independent factors.

Variables	Multivariate analysis		
	OR	95% CI	*P*-value
Age	1.057	1.030 – 1.085	<0.001
Urea	1.050	1.011 – 1.092	0.013
Na	1.057	1.003 – 1.113	0.037
MCHC	0.990	0.981 – 0.999	0.038
APTT	1.048	1.018 – 1.079	0.002

AUC, Area Under the Curve; Age, Age; APTT, Activated Partial Thromboplastin Time; OR, Odds Ratio; CI, Confidence Interval; MCHC, Mean Corpuscular Hemoglobin Concentration; Na, Sodium; Urea, Urea.

### Development and validation of the nomogram

Based on the multivariate logistic regression analysis, a nomogram was developed to predict 30-day mortality (**Figure 3A**). ROC analysis demonstrated an AUC of 0.813 (95% CI: 0.749–0.877) in the training cohort and 0.808 (95% CI: 0.714–0.902) in the validation cohort (**Figure 3B**). The calibration curves showed excellent agreement between the predicted probabilities and the actual outcomes of 30-day mortality in both cohorts (**Figures 3C, D**). Furthermore, decision curve analysis (DCA) indicated that using the model to predict 30-day mortality provided a substantial net benefit across a wide range of threshold probabilities in both cohorts, supporting its potential clinical utility (**Figures 3E, F**).

**Figure 3:**
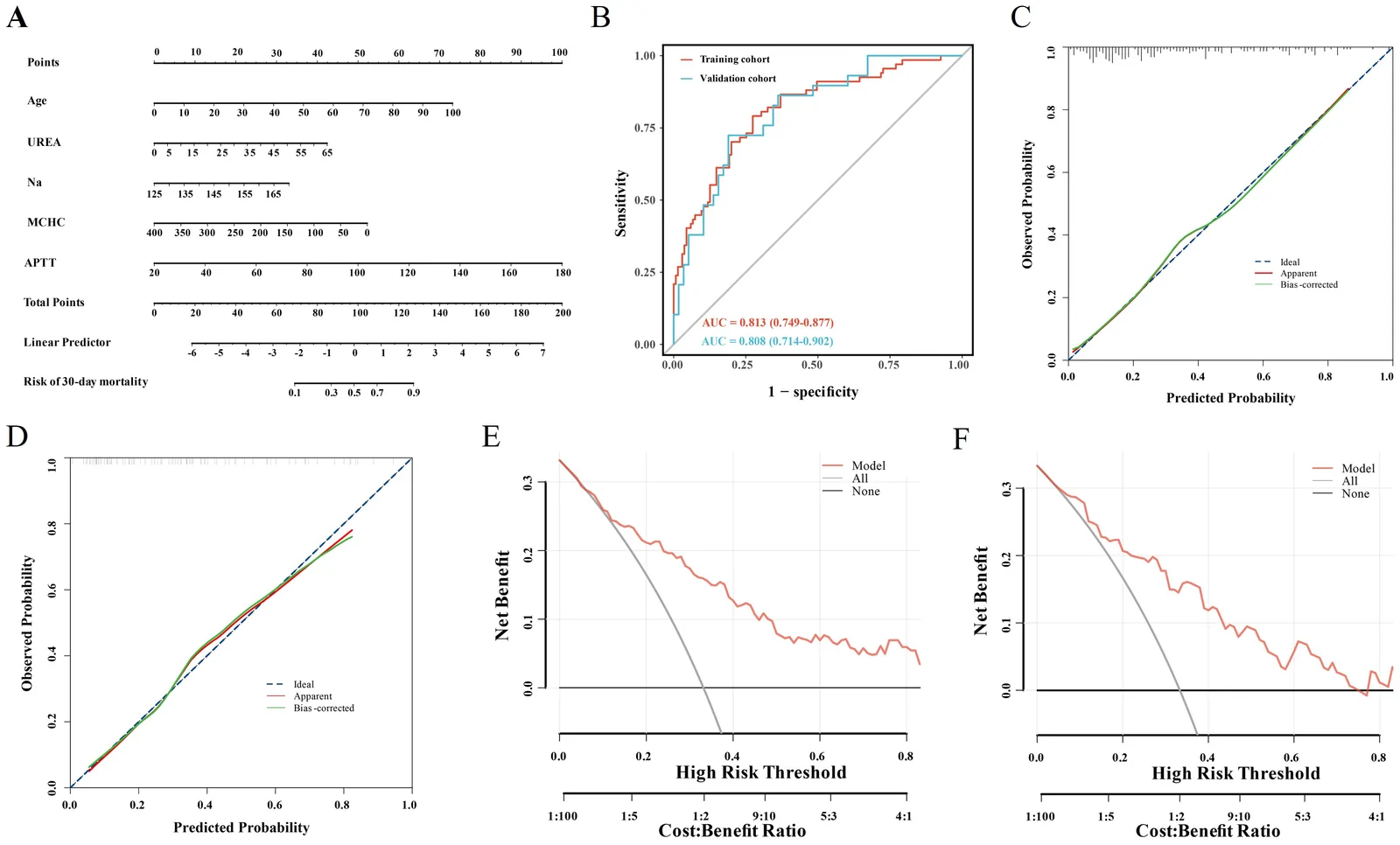
Performance of the nomogram. **(A)** Nomogram **(B)** ROC curves for 30-day mortality. **(C)** Calibration curves in the training cohort. **(D)** Calibration curves in the validation cohort. **(E)** DCA in the training cohort. **(F)** DCA in the validation cohort.

## Discussion

*A. Baumannii* is a common opportunistic pathogen responsible for hospital-acquired infections, accounting for approximately 6%–7% of such infections in China, particularly among critically ill patients (Wang et al., 2024). *A. baumannii* can cause various severe infections, with ventilator-associated pneumonia (VAP) and central catheter-related bloodstream infections (CRBSIs) being among the most life-threatening (Weiner et al., 2016). A multicenter cohort study across 24 countries identified *A. baumannii* as the most common causative agent of hospital-acquired BSI (Tabah et al., 2012). Despite a relatively low isolation rate, *A. baumannii*-associated BSI carries a high mortality rate exceeding 60% (Global mortality associated with 33 bacterial pathogens in 2019: a systematic analysis for the Global Burden of Disease Study 2019, 2022). For example, an observational study across 12 tertiary hospitals in Italy reported a 30-day mortality rate of 73.6% for such infections (Russo et al., 2019).

The growing prevalence of carbapenem-resistant *Acinetobacter baumannii* (CRAB) further complicates management. Data from the China Antimicrobial Resistance Surveillance Network (CHINET) show that resistance rates to meropenem and imipenem rose from 30.1% and 39.0% in 2005 to 71.5% and 72.3% in 2021, respectively (Yang et al., 2012; Li et al., 2019). This trend poses significant challenges to healthcare systems and imposes a substantial economic burden.

Given the widespread drug resistance and high mortality associated with *A. baumannii* BSI, identifying prognostic factors is clinically imperative. In this retrospective study, we used LASSO regression and the Boruta algorithm, followed by multivariate logistic regression, to identify risk factors for 30-day mortality from sex, age, and all laboratory parameters. Age, Urea, Na, MCHC and APTT were identified as independent risk factors. A nomogram was developed to predict 30-day mortality risk. The AUC was 0.813 in the training cohort and 0.808 in the validation cohort. Calibration curves showed good agreement between predicted and observed outcomes, and DCA indicated a positive net clinical benefit. These results suggested that the nomogram demonstrated satisfactory predictive performance, good calibration, and potential clinical utility.

Several previous studies have developed prognostic models for *A. baumannii* BSI, but most rely on clinical variables that require comprehensive assessment (e.g., APACHE II scores, mechanical ventilation, septic shock, organ failure) or pathogen-specific data (e.g., carbapenem resistance) that may not be available at the time of blood culture collection (Zhang et al., 2020; Song et al., 2023; Li et al., 2024). In contrast, our study intentionally focused on readily available demographic (specifically age) and routine laboratory parameters to develop a simplified predictive tool. This design choice enhances the model’s early-stage applicability and potential generalizability, as these variables are routinely available at the time of blood culture collection without requiring complex clinical scoring systems. Despite using fewer and simpler variables, our model achieved an AUC of 0.813 (training) and 0.808 (validation), comparable to the performance of prior models that incorporated more complex clinical predictors. It is worth noting that our model is not intended to replace comprehensive clinical assessment or established severity scores; rather, it serves as an early adjunct that can later be supplemented by pathogen-specific and clinical data as they become available.

In our study, older age was a significant predictor of 30-day mortality in patients with *A. baumannii* BSI. This aligns with previous research. A retrospective study in Saudi Arabia (2015–2022) of 122 patients with *A. baumannii* BSI found that age ≥65 years was significantly associated with 90-day mortality (P<0.05). Notably, the carbapenem-resistant *A. baumannii* (CRAB) isolation rate in this cohort was 69.6%, with a mortality rate of 74.4% in the CRAB-infected subgroup (Kaki, 2024). Similarly, Guo et al., in a study of 191 patients with CRAB BSI (2018–2023), confirmed that advanced age (OR = 1.03, p = 0.02), mechanical ventilation, and septic shock were independent predictors of 30-day mortality (Guo et al., 2016). The underlying mechanism may involve immunosenescence and chronic inflammation associated with aging, which lead to impaired neutrophil function (Van Avondt et al., 2023), dysregulated cytokine secretion (e.g., elevated interleukin-6 [IL-6]) (Li et al., 2025), and defective T cell function (Inoue et a., 2014). Our findings corroborate that age is an independent risk factor for 30-day mortality in this patient population. Juttukonda et al. found that *A. baumannii* utilizes urea as its sole nitrogen source and its metabolites (e.g., ammonia), can regulate the local microenvironmental pH, thereby promoting biofilm formation and immune evasion (Juttukonda et al., 2016). Furthermore, the bacterium resists host calprotectin-mediated chelation of zinc and manganese ions through synergistic interactions between urea metabolism genes (e.g., *mumA*, *mumB*, *mumC*) and metal transport systems, enhancing its survival in the host environment (Damo et al., 2013). Supporting the role of urea metabolism in virulence, strains with knocked-out urea metabolism genes exhibited significantly reduced colonization capacity in a murine pneumonia model (Juttukonda et al., 2016). Clinically, Feng et al. found that the blood urea nitrogen (BUN)-to-albumin ratio was significantly associated with in-hospital mortality in patients with *A. baumannii* hospital-acquired pneumonia (Feng et al., 2022). Our finding that elevated blood urea is an independent risk factor for 30-day mortality in *A. baumannii* BSI is thus consistent with both pathogenic mechanisms and clinical evidence.

Hypernatremia, defined as a plasma sodium concentration exceeding 145 mmol/L (Ilardi, 2022), induces a hypertonic state with profound systemic effects, including cerebral atrophy, demyelination, rhabdomyolysis, and impaired cardiac function (Mastro-Martínez et al., 2015; Rugg et al., 2020). In our cohort, non-survivors with *A. baumannii* BSI had significantly higher serum sodium levels than survivors. This aligns with studies in broader sepsis populations. Ni et al., in a retrospective study of 51 ICU patients with sepsis, confirmed that hypernatremia (Na^+^ > 145 mmol/L) was significantly associated with mortality (OR = 1.15, 95% CI: 1.022–1.294) and prolonged hospitalization (Ni et al., 2016). Similarly, Shirazy et al. found that hypernatremia on day 7 after admission was an independent predictor of mortality in septic patients (OR = 1.26, p = 0.039) (Shirazy et al., 2020). Furthermore, hypernatremia has been linked to an increased risk of bloodstream infection itself, as demonstrated in a cohort of 823 dialysis patients (Lo et al., 2022). The detrimental effects of hypernatremia may be mediated by osmotic stress, which causes cellular dehydration and activates inflammatory pathways such as nuclear factor kappa B (NF-κB) and mitogen-activated protein kinase (MAPK). This activation promotes the release of pro-inflammatory cytokines (e.g., IL-6, TNF-α), potentially creating a vicious cycle of inflammation and hypernatremia (Shi et al., 2025). Additionally, dehydration of neurons, endothelial cells, and renal tubular cells can lead to acute kidney injury and microcirculatory dysfunction, progressing to multiple organ dysfunction syndrome (MODS) (Fang and Xie, 2025). Therefore, early detection of hypernatremia and careful fluid management (e.g., correcting water deficits, restricting sodium intake) may improve outcomes for patients with *A. baumannii* BSI.

MCHC is calculated as the ratio of HGB concentration to HCT (Girard et al., 2025), reflects the hemoglobin concentration within red blood cells (RBCs) and is associated with anemia subtypes (e.g., iron deficiency anemia, megaloblastic anemia) (Gupta et al., 2003; Azarkhish et al., 2012). Abnormal MCHC levels may affect the oxygen-carrying capacity (Turner et al., 1987) and immune function of RBC (Kumar et al., 2022). Higher MCHC levels may indicate improved oxygen delivery to tissues, potentially supporting organ function and survival. MCHC is also considered an inflammatory marker that can influence prognosis (Lippi et al., 2009), as inflammation can induce iron deficiency (McClung and Karl, 2009), which in turn may lower MCHC levels (Archer and Brugnara, 2015). Supporting its prognostic value, Chen et al., in a retrospective multicenter cohort study using the MIMIC-IV and eICU-CRD databases, found that higher MCHC levels were associated with reduced 30- and 90-day mortality in critically ill ICU patients with acute kidney injury (AKI) (Chen et al., 2025). Similarly, Lin et al., using the MIMIC-IV database, reported that decreased MCHC was associated with in-hospital mortality in patients with liver cirrhosis complicated by sepsis (Lin et al., 2024). Thus, MCHC has emerged as a prognostic marker in various conditions. Consistent with this, our findings showed that MCHC levels were significantly higher in survivors than in non-survivors of *A. baumannii* BSI.

Coagulation dysfunction during *A. baumannii* BSI can influence clinical presentation and prognosis. APTT is a key screening test for intrinsic coagulation pathway disorders and typically prolongs with coagulation factor deficiencies or dysfunction. In an outbreak investigation of *A. baumannii* BSI in a Saudi Arabian neonatal intensive care unit (NICU), APTT was significantly prolonged (p=0.001) in seven diagnosed infants, five of whom exhibited features of disseminated intravascular coagulation (DIC). Prolonged APTT was directly associated with thrombocytopenia (p=0.001), decreased hemoglobin (p=0.0001), and increased mortality (El-Feky and Aref, 2011). Mechanistically, *in vitro* studies by the U.S. National Institutes of Health (NIH) have shown that CpaA, a zinc-dependent metallo-endopeptidase secreted by clinical *A. baumannii* strains, degrades coagulation factor V and fibrinogen, leading to prolonged APTT (p<0.001) and reduced thrombin production (Tilley et al., 2014). This mechanism may explain the observed coagulation dysfunction in patients. Therefore, prolonged APTT in *A. baumannii* BSI may reflect infection severity and organ failure risk. Combining APTT with other indicators (e.g., platelet count) could aid in prognostic assessment and guide clinical management.

In summary, we developed a nomogram to predict the 30-day mortality risk for patients with *A. baumannii* BSI. All enrolled patients had microbiologically confirmed *A. baumannii* BSI, and the model was developed and internally validated exclusively in this population. The five predictors are not pathogen-specific and primarily reflect general physiological status or organ dysfunction. This was a deliberate design choice: the model is intended for early risk stratification once *A. baumannii* BSI has been confirmed but before susceptibility results are available, although whether it can guide empirical antibiotic selection has not been validated in this study and requires further prospective investigation.

However, this study had several limitations. First, it was a single-center retrospective analysis with a relatively small sample size, which may have introduced selection bias and limited generalizability. Second, important treatment-related confounders, including prior antibiotic exposure and time to effective antimicrobial therapy, were not systematically available and could not be included in the model. Third, our model intentionally excluded clinical variables to maintain early applicability, and although MDR infection status was included as a candidate variable, it did not enter the final model. These factors may limit the model’s performance. Fourth, laboratory data were collected only at a single time point, and dynamic changes in parameters were not captured. Therefore, our conclusions apply only to confirmed *A. baumannii* BSI, and validation in larger, multi-center prospective studies is required.

## Conclusions

In conclusion, we identified age, Urea, Na, MCHC, and APTT as factors closely associated with prognosis in patients with *A. baumannii* BSI. The developed predictive model based on age and routine laboratory parameters, is intended for early risk stratification after confirmation of *A. baumannii* BSI but before antimicrobial susceptibility results are available. It should be regarded as a tool to support early risk assessment rather than to guide empirical antibiotic selection, and its clinical utility requires further prospective validation.

## Data Availability

The raw data supporting the conclusions of this article will be made available by the authors, without undue reservation.
