# Supplementary material for: A nomogram for early risk stratification of 30-day mortality in confirmed Acinetobacter baumannii bloodstream infection
Source: Front Cell Infect Microbiol. 2026 Oct 30;16:1979847. doi: 10.3389/fcimb.2026.1979847 (PMC13759356; doi:10.3389/fcimb.2026.1979847)
Supplement: Supplementary file 1 [file Table1.docx]

**Supplementary Table 1.**Baseline characteristics and laboratory parameter.

| **Characteristic** | **Training Cohort** | | | | **Validation Cohort  N = 87** | **P_2-_value** |
| --- | --- | --- | --- | --- | --- | --- |
|  | **Overall**  **N = 202** | **Survivor  N = 135** | **Non-survivor**  **N = 67** | **P_1_-value** |  |  |
| **Gender(male), n (%)** | 132 (65.3%) | 84 (62.2%) | 48 (71.6%) | 0.185 | 62 (71.3%) | 0.326 |
| **Age, y, M (Q1, Q3)** | 63 (50, 70) | 57 (49, 66) | 69 (57, 77) | <0.001 | 66 (50, 73) | 0.102 |
| **Hypertension, n (%)** | 86 (42.6%) | 53 (39.3%) | 33 (49.3%) | 0.176 | 38 (43.7%) | 0.862 |
| **Diabetes, n (%)** | 46 (22.8%) | 24 (17.8%) | 22 (32.8%) | 0.016 | 18 (20.7%) | 0.696 |
| **Coronary heart disease, n (%)** | 27 (13.4%) | 13 (9.6%) | 14 (20.9%) | 0.046 | 13 (14.9%) | 0.349 |
| **Respiratory diseases, n (%)** | 5 (2.5%) | 1 (0.7%) | 4 (6.0%) | 0.042 | 5 (5.7%) | 0.174 |
| **Cerebrovascular disease, n (%)** | 20 (9.9%) | 11 (8.1%) | 9 (13.4%) | 0.236 | 10 (11.5%) | 0.684 |
| **Chronic renal disease, n (%)** | 9 (4.5%) | 5 (3.7%) | 4 (6.0%) | 0.483 | 2 (2.3%) | 0.514 |
| **Hematological Disease, n (%)** | 1 (0.5%) | 0 (0.0%) | 1 (1.5%) | 0.332 | 3 (3.4%) | 0.083 |
| **Autoimmune diseases, n (%)** | 7 (3.5%) | 7 (5.2%) | 0 (0.0%) | 0.098 | 2 (2.3%) | 0.728 |
| **Malignancy, n (%)** | 26 (12.9%) | 15 (11.1%) | 11 (16.4%) | 0.289 | 8 (9.2%) | 0.374 |
| **Smoking history, n (%)** | 44 (21.8%) | 28 (20.7%) | 16 (23.9%) | 0.611 | 18 (20.7%) | 0.836 |
| **Alcohol history, n (%)** | 36 (17.8%) | 25 (18.5%) | 11 (16.4%) | 0.713 | 10 (11.5%) | 0.177 |
| **ICU admission status, n (%)** | 120 (59.4%) | 75 (55.6%) | 45 (67.2%) | 0.114 | 46 (52.9%) | 0.303 |
| **Blood transfusion history, n (%)** | 120 (59.4%) | 77 (57.0%) | 43 (64.2%) | 0.330 | 44 (50.6%) | 0.165 |
| **Surgical history, n (%)** | 109 (54.0%) | 80 (59.3%) | 29 (43.3%) | 0.032 | 46 (52.9%) | 0.865 |
| **Invasive mechanical ventilation, n (%)** | 128 (63.4%) | 73 (54.1%) | 55 (82.1%) | <0.001 | 46 (52.9%) | 0.095 |
| **Arterial catheterization, n (%)** | 14 (6.9%) | 8 (5.9%) | 6 (9.0%) | 0.557 | 0 (0.0%) | 0.007 |
| **Venous catheterization, n (%)** | 68 (33.7%) | 38 (28.1%) | 30 (44.8%) | 0.019 | 29 (33.3%) | 0.957 |
| **Drainage tubes, n (%)** | 75 (37.1%) | 53 (39.3%) | 22 (32.8%) | 0.374 | 31 (35.6%) | 0.809 |
| **Urinary catheters, n (%)** | 24 (11.9%) | 17 (12.6%) | 7 (10.4%) | 0.657 | 11 (12.6%) | 0.855 |
| **Gastrointestinal catheters, n (%)** | 20 (9.9%) | 17 (12.6%) | 3 (4.5%) | 0.069 | 7 (8.0%) | 0.619 |
| **Renal replacement therapy, n (%)** | 59 (29.2%) | 27 (20.0%) | 32 (47.8%) | <0.001 | 26 (29.9%) | 0.908 |
| **Septic shock, n (%)** | 61 (30.2%) | 23 (17.0%) | 38 (56.7%) | <0.001 | 23 (26.4%) | 0.518 |
| **Organ failure, n (%)** | 40 (19.8%) | 9 (6.7%) | 31 (46.3%) | <0.001 | 18 (20.7%) | 0.863 |
| **Using hormones/ immunosuppressants, n (%)** | 78 (38.6%) | 34 (25.2%) | 44 (65.7%) | <0.001 | 35 (40.2%) | 0.796 |
| **Multidrug resistant bacterial infection n (%)** | 151 (74.8%) | 93 (68.9%) | 58(86.6%) | 0.006 | 62(71.3%) | 0.537 |
| **CRP (mg/L), M (Q1, Q3)** | 56 (35, 89) | 55 (33, 86) | 60 (36, 90) | 0.179 | 60 (31, 90) | 0.933 |
| **WBC (×10^9^/L), M (Q1, Q3)** | 9 (5, 13) | 9 (5, 13) | 9 (5, 15) | 0.781 | 8 (5, 17) | 0.593 |
| **LY count (×10^9^/L), M (Q1, Q3)** | 0.63 (0.36, 1.06) | 0.69 (0.40, 1.12) | 0.50 (0.26, 0.97) | 0.083 | 0.61 (0.30, 0.97) | 0.733 |
| **MO count (×10^9^/L), M (Q1, Q3)** | 0.43 (0.15, 0.72) | 0.43 (0.20, 0.73) | 0.41 (0.10, 0.72) | 0.511 | 0.40 (0.12, 0.69) | 0.687 |
| **NE count (×10^9^/L), M (Q1, Q3)** | 7.3 (4.0, 11.7) | 7.3 (3.9, 10.6) | 7.3 (4.0, 12.1) | 0.882 | 7.2 (3.6, 13.6) | 0.638 |
| **EO count (×10^9^/L), M (Q1, Q3)** | 0.03 (0.00, 0.10) | 0.04 (0.01, 0.12) | 0.01 (0.00, 0.06) | 0.012 | 0.02 (0.00, 0.08) | 0.376 |
| **BA count (×10^9^/L), M (Q1, Q3)** | 0.020 (0.010, 0.040) | 0.020 (0.010, 0.040) | 0.010 (0.010, 0.030) | 0.521 | 0.020 (0.010, 0.030) | 0.997 |
| **LY percentage (%), M (Q1, Q3)** | 7 (4, 14) | 7 (4, 14) | 7 (3, 15) | 0.585 | 6 (4, 14) | 0.579 |
| **MO percentage (%), M (Q1, Q3)** | 4.9 (2.2, 7.5) | 5.3 (2.6, 7.5) | 4.1 (2.0, 7.3) | 0.372 | 4.5 (2.0, 6.8) | 0.253 |
| **NE percentage (%), M (Q1, Q3)** | 85 (76, 91) | 84 (77, 91) | 87 (75, 91) | 0.541 | 86 (79, 92) | 0.461 |
| **EO percentage (%), M (Q1, Q3)** | 0.30 (0.00, 1.20) | 0.40 (0.10, 1.40) | 0.20 (0.00, 0.92) | 0.033 | 0.27 (0.00, 1.00) | 0.489 |
| **BA percentage (%), M (Q1, Q3)** | 0.20 (0.10, 0.40) | 0.20 (0.10, 0.40) | 0.20 (0.10, 0.36) | 0.897 | 0.20 (0.10, 0.30) | 0.838 |
| **RBC (×10^12^/L), M (Q1, Q3)** | 3.06 (2.67, 3.60) | 3.19 (2.71, 3.69) | 2.91 (2.59, 3.27) | 0.016 | 2.89 (2.42, 3.35) | 0.036 |
| **HGB (g/L), M (Q1, Q3)** | 95 (81, 108) | 98 (82, 112) | 87 (79, 99) | 0.014 | 89 (78, 103) | 0.092 |
| **HCT (%), M (Q1, Q3)** | 29 (25, 33) | 30 (25, 34) | 28 (24, 32) | 0.088 | 27 (24, 31) | 0.038 |
| **MCV (fl), M (Q1, Q3)** | 93.4 (88.9, 97.1) | 92.5 (88.6, 95.9) | 94.1 (89.6, 98.1) | 0.047 | 93.0 (91.2, 97.3) | 0.290 |
| **MCH (pg), M (Q1, Q3)** | 30.40 (29.50, 31.40) | 30.30 (29.60, 31.50) | 30.40 (29.40, 31.40) | 0.806 | 30.60 (29.80, 32.10) | 0.083 |
| **MCHC (g/L), M (Q1, Q3)** | 327 (317, 336) | 329 (321, 337) | 321 (309, 331) | <0.001 | 328 (316, 338) | 0.582 |
| **RDW (%), M (Q1, Q3)** | 14.80 (13.50, 16.40) | 14.40 (13.30, 16.30) | 15.58 (14.40, 16.70) | 0.002 | 15.20 (13.90, 17.20) | 0.112 |
| **PLT(×10^9^/L), M (Q1, Q3)** | 129 (63, 200) | 140 (77, 232) | 88 (47, 151) | <0.001 | 123 (68, 173) | 0.882 |
| **PCT (%), M (Q1, Q3)** | 0.15 (0.10, 0.24) | 0.17 (0.11, 0.24) | 0.11 (0.07, 0.22) | 0.036 | 0.14 (0.10, 0.21) | 0.826 |
| **MPV (fl), M (Q1, Q3)** | 11.50 (10.50, 12.40) | 11.20 (10.20, 12.30) | 11.90 (11.00, 12.50) | 0.016 | 11.70 (10.40, 12.50) | 0.322 |
| **PDW (%), M (Q1, Q3)** | 16.0 (13.2, 17.1) | 15.7 (12.6, 16.8) | 16.2 (14.4, 17.2) | 0.052 | 15.7 (13.6, 17.1) | 0.943 |
| **Pro-BNP (pg/mL), M (Q1, Q3)** | 2,617 (706, 7,497) | 2,237 (554, 5,541) | 4,229 (872, 10,787) | 0.003 | 2,401 (723, 6,619) | 0.850 |
| **PCT (ng/mL), M (Q1, Q3)** | 2 (0, 8) | 2 (0, 8) | 4 (1, 11) | 0.039 | 2 (0, 6) | 0.250 |
| **PT (s), M (Q1, Q3)** | 14.3 (12.7, 17.1) | 13.7 (12.6, 15.9) | 16.4 (13.8, 19.4) | <0.001 | 14.2 (13.3, 15.9) | 0.669 |
| **PT-INR, M (Q1, Q3)** | 1.29 (1.13, 1.59) | 1.22 (1.11, 1.43) | 1.51 (1.25, 1.85) | <0.001 | 1.27 (1.18, 1.47) | 0.741 |
| **APTT (s), M (Q1, Q3)** | 34 (29, 44) | 31 (29, 38) | 41 (32, 54) | <0.001 | 34 (30, 41) | 0.967 |
| **FIB (g/L), M (Q1, Q3)** | 3.19 (2.16, 4.58) | 3.42 (2.38, 4.73) | 2.46 (1.88, 3.77) | 0.003 | 3.36 (1.96, 4.84) | 0.655 |
| **TT (s), M (Q1, Q3)** | 16.8 (15.6, 18.3) | 16.6 (15.6, 17.8) | 17.5 (15.8, 19.2) | 0.037 | 17.3 (15.7, 18.7) | 0.487 |
| **D-dimer (mg/L), M (Q1, Q3)** | 5 (2, 9) | 4 (2, 8) | 5 (2, 11) | 0.142 | 4 (2, 9) | 0.650 |
| **ALT (U/L), M (Q1, Q3)** | 27 (15, 54) | 27 (16, 60) | 24 (13, 41) | 0.216 | 25 (14, 53) | 0.787 |
| **AST (U/L), M (Q1, Q3)** | 36 (23, 59) | 35 (24, 58) | 41 (22, 72) | 0.642 | 37 (24, 80) | 0.697 |
| **ALP (U/L), M (Q1, Q3)** | 116 (72, 177) | 121 (74, 187) | 98 (69, 155) | 0.149 | 113 (73, 164) | 0.964 |
| **GGT (U/L), M (Q1, Q3)** | 59 (29, 163) | 67 (32, 192) | 44 (23, 103) | 0.017 | 68 (28, 126) | 0.779 |
| **LDH (U/L), M (Q1, Q3)** | 320 (236, 417) | 302 (220, 383) | 331 (267, 534) | 0.003 | 300 (220, 421) | 0.704 |
| **CK (U/L), M (Q1, Q3)** | 53 (27, 162) | 52 (27, 142) | 59 (25, 270) | 0.623 | 61 (27, 218) | 0.524 |
| **HBDH (U/L), M (Q1, Q3)** | 197 (147, 268) | 190 (127, 252) | 216 (169, 351) | 0.001 | 186 (124, 280) | 0.306 |
| **TBIL (μmol/L), M (Q1, Q3)** | 17 (11, 33) | 17 (10, 27) | 19 (12, 41) | 0.147 | 16 (10, 31) | 0.540 |
| **DBIL (μmol/L), M (Q1, Q3)** | 9 (5, 19) | 8 (5, 18) | 12 (7, 27) | 0.078 | 8 (5, 21) | 0.687 |
| **IBIL (μmol/L), M (Q1, Q3)** | 7 (5, 12) | 7 (5, 12) | 8 (5, 14) | 0.424 | 7 (5, 11) | 0.361 |
| **TC (mmol/L), M (Q1, Q3)** | 2.85 (1.78, 3.77) | 2.97 (2.09, 3.94) | 2.03 (1.46, 3.16) | <0.001 | 2.53 (1.78, 3.33) | 0.394 |
| **TG (mmol/L), M (Q1, Q3)** | 1.28 (0.89, 1.92) | 1.27 (0.89, 1.88) | 1.33 (0.89, 2.09) | 0.646 | 1.43 (0.84, 2.33) | 0.358 |
| **HDL-C(mmol/L), M (Q1, Q3)** | 0.59 (0.38, 0.83) | 0.65 (0.41, 0.90) | 0.53 (0.29, 0.72) | 0.005 | 0.53 (0.36, 0.77) | 0.251 |
| **LDL-C(mmol/L), M (Q1, Q3)** | 1.86 (1.11, 2.57) | 2.01 (1.26, 2.59) | 1.47 (0.85, 2.23) | 0.005 | 1.56 (1.15, 2.22) | 0.260 |
| **TP (g/L), Mean ± SD** | 58 ± 9 | 59 ± 8 | 55 ± 9 | 0.001 | 58 ± 7 | 0.776 |
| **ALB (g/L), Mean ± SD** | 33.3 ± 5.6 | 34.0 ± 5.5 | 31.8 ± 5.5 | 0.010 | 34.2 ± 6.0 | 0.250 |
| **GLB (g/L), M (Q1, Q3)** | 24 (20, 29) | 25 (21, 30) | 23 (18, 27) | 0.044 | 23 (20, 28) | 0.466 |
| **A/G, M (Q1, Q3)** | 1.40 (1.10, 1.70) | 1.40 (1.10, 1.80) | 1.40 (1.10, 1.70) | 0.538 | 1.40 (1.10, 1.90) | 0.391 |
| **GLU (mmol/L), M (Q1, Q3)** | 7.3 (5.4, 9.5) | 7.1 (5.2, 9.3) | 8.1 (5.8, 11.5) | 0.038 | 6.9 (5.1, 11.1) | 0.899 |
| **Urea (mmol/L), M (Q1, Q3)** | 9 (6, 16) | 8 (6, 13) | 13 (8, 23) | <0.001 | 11 (7, 19) | 0.231 |
| **Cr (μmol/L), M (Q1, Q3)** | 69 (49, 134) | 64 (48, 96) | 93 (52, 188) | 0.008 | 80 (55, 143) | 0.198 |
| **UA(μmol/L), M (Q1, Q3)** | 192 (116, 286) | 188 (105, 270) | 196 (119, 334) | 0.263 | 214 (144, 309) | 0.255 |
| **Ca (mmol/L), M (Q1, Q3)** | 2.13 (2.01, 2.25) | 2.16 (2.02, 2.26) | 2.09 (1.99, 2.21) | 0.093 | 2.12 (2.01, 2.24) | 0.876 |
| **P (mmol/L), M (Q1, Q3)** | 0.99 (0.77, 1.26) | 1.02 (0.80, 1.32) | 0.91 (0.67, 1.14) | 0.033 | 1.02 (0.84, 1.20) | 0.795 |
| **Mg(mmol/L), M (Q1, Q3)** | 0.87 (0.78, 0.95) | 0.87 (0.78, 0.93) | 0.86 (0.79, 0.97) | 0.799 | 0.87 (0.80, 0.95) | 0.679 |
| **K (mmol/L), M (Q1, Q3)** | 3.99 (3.60, 4.41) | 3.98 (3.62, 4.40) | 4.02 (3.56, 4.49) | 0.585 | 3.93 (3.71, 4.35) | 0.979 |
| **Na (mmol/L), M (Q1, Q3)** | 140 (137, 144) | 140 (136, 142) | 143 (140, 146) | <0.001 | 140 (137, 144) | 0.722 |
| **Cl (mmol/L), M (Q1, Q3)** | 103 (100, 108) | 103 (100, 107) | 103 (100, 110) | 0.768 | 105 (100, 109) | 0.351 |
| **RBP (mg/L), M (Q1, Q3)** | 23 (15, 35) | 23 (14, 36) | 24 (15, 35) | 0.905 | 27 (18, 39) | 0.090 |
| **ADA(U/L), M (Q1, Q3)** | 11 (8, 14) | 10.9 (7.9, 14.2) | 10.8 (6.5, 14.0) | 0.452 | 11 (8, 13) | 0.947 |

ADA: Adenosine Deaminase; ALB: Albumin; ALP: Alkaline Phosphatase; ALT: Alanine Aminotransferase; APTT: Activated Partial Thromboplastin Time; AST: Aspartate Aminotransferase; A/G: Albumin-to-Globulin Ratio; BA: Basophils; Ca: Calcium; CK: Creatine Kinase; Cl: Chloride; CRP: C-Reactive Protein; CREA: Creatinine; DBIL: Direct Bilirubin; EO: Eosinophils; FIB: Fibrinogen; GGT: Gamma-Glutamyl Transferase; GLB: Globulin; GLU: Glucose; HBDH: Hydroxybutyrate Dehydrogenase; HCT: Hematocrit; HGB: Hemoglobin; HDL-C: High-Density Lipoprotein Cholesterol; IBIL: Indirect Bilirubin; ICU: Intensive Care Unit; K: Potassium; LDH: Lactate Dehydrogenase; LDL-C: Low-Density Lipoprotein Cholesterol; LY: Lymphocytes; M (Q1, Q3): Median (First Quartile, Third Quartile); MCH: Mean Corpuscular Hemoglobin; MCHC: Mean Corpuscular Hemoglobin Concentration; MCV: Mean Corpuscular Volume; Mg: Magnesium; MO: Monocytes; MPV: Mean Platelet Volume;
Na: Sodium; NE: Neutrophils; P: Phosphorus; PDW: Platelet Distribution Width; PCT: Procalcitonin; PCT: Plateletcrit; PLT: Platelets; Pro-BNP: Pro-B-type Natriuretic Peptide; PT: Prothrombin Time; PT-INR: Prothrombin Time International Normalized Ratio; RBC: Red Blood Cell Count; RBP: Retinol-Binding Protein; RDW: Red Cell Distribution Width; SD: Standard Deviation; TBIL: Total Bilirubin; TC: Total Cholesterol; TG: Triglycerides; TP: Total Protein; TT: Thrombin Time; UA: Uric Acid; WBC: White Blood Cell Count.
